# Comment on: Diagnostic value of miR-186-5p for carotid artery stenosis and its predictive significance for future cerebral ischemic event

**DOI:** 10.1186/s13000-020-01055-2

**Published:** 2020-11-22

**Authors:** Siamak Sabour

**Affiliations:** 1 Department of Clinical Epidemiology, School of Health and Safety, Shahid Beheshti University of Medical Sciences, Tehran, IR Iran; 2 Safety Promotions and Injury Prevention Research Centre, Shahid Beheshti University of Medical Sciences, Tehran, IR Iran

**Keywords:** Asymptomatic CAS, Cerebral ischemic events, Diagnosis, MiR-186-5p

I read the article by Lv et al. published in Diagn Pathol 2020. They aimed to investigate the diagnostic value of miR-186-5p for asymptomatic carotid artery stenosis (CAS), and its predictive value for future cerebral ischemic events (CIEs) [1]. Sixty-seven cases with asymptomatic CAS and 60 healthy individuals were recruited. Receiving-operator characteristic (ROC) curve was drawn based on sensitivity and specificity analyses. Kaplan-Meier method was applied for the evaluation of the predictive value of miR-186-5p for the occurrence of CIE. They reported that area under the curve (AUC) was 0.91, with the sensitivity of 89.6% and specificity of 81.7% at the cutoff value of 1.22. Kaplan-Meier method results revealed that high miR-186-5p level was associated with the occurrence of CIEs.

While the article offers insight into the decision that MiR-186-5p is a possible diagnostic biomarker for patients with asymptomatic CAS and predicts the incidence of future CIEs, its conclusion is restricted in three ways. First,knowledge of the reported estimates does not provide overall information on the diagnostic and prognostic value of MiR-186-5p in clinical practice. Diagnostic added value is much more important for clinical purposes than the estimates stated [2–5]. Diagnostic knowledge is the information required to address the issue, “What is the possibility of the presence or absence of a particular disease given these test results? “ (Research for diagnostic accuracy).

Therefore, diagnostic added value of MiR-186-5p (differences of ROC curves for two diagnostic models with and withour MiR-186-5p) is greatly important in clinical practice. Diagnostic added value of MiR-186-5p may indeed be minimal, although validity estimates may still be excellent. On the other hand, I should mention that as high miR-186-5p level and high degree of carotid stenosis were independent factors for the occurrence of CIEs, one might consider the specificity of these two factors be combined. Combination of the tests are common in clinical practice which increase the specificity and allows to rule in a diagnosis. Second, without determining reliability (precision), we can not judge the diagnostic value of MiR-186-5p. The diagnostic value is determined by the following two parameters: calibration (reliability) and discrimination (accuracy) [2, 6–8].

Third, for medical purposes, the global average approach (Kaplan-Meier method) can not be used to predict of an individual based outcome. In addition, for prediction of CIEs, we require data from two separate cohorts or at least one cohort split into two to first build a prediction model and then test our prediction model. Misleading outcomes are generally the main outcome of the research that fails to test the prediction models [9–11]. Therefore, reporting association (HR = 4.1) even statistically significant do not gurrantee correct prediction.

I therefore claim that there are certain technical weaknesses and strategies to fix them when determining the predictive and diagnostic value of MiR-186-5p; otherwise, misinterpretation can not be eliminated.

## Data Availability

Not applicable.

## Abbreviations

miRNAs: MicroRNAs
CAS: Carotid artery stenosis
CIEs: Cerebral ischemic events
ROC: Receiving–operator characteristic
HRs: Hazard ratios
AUC: Under the curve
